# Uncertainties of recalculated bond lengths, angles and polyhedral volumes as implemented in the *Crystal Palace* program for parametric crystal structure analysis

**DOI:** 10.1107/S2053273325002682

**Published:** 2025-04-29

**Authors:** Ross J. Angel, Mattia L. Mazzucchelli, Lisa Baratelli, Catherine F. Schweinle, Tonci Balić-Žunić, Javier Gonzalez-Platas, Matteo Alvaro

**Affiliations:** a Istituto di Geoscienze e Georisorse Consiglio Nazionale delle Ricerche Corso Stati Uniti 4 Padova PD35127 Italy; b Institute of Earth Sciences University of Lausanne UNIL-Mouline Lausanne CH-1015 Switzerland; c Department of Earth and Environmental Sciences University of Pavia Via A. Ferrata, 1 Pavia PV27100 Italy; d Institute for Inorganic and Analytical Chemistry, and Freiburg Materials Research Center (FMF) Albert-Ludwigs-University Freiburg Freiburg Germany; e Department of Geosciences and Natural Resource Management University of Copenhagen Øster Voldgade 10 Copenhagen Denmark; f Departamento de Física, Instituto Universitario de Estudios Avanzados en Física Atómica, Molecular y Fotónica (IUDEA), MALTA Consolider Team Universidad de La Laguna La Laguna Tenerife38204 Spain; Institute of Crystallography - CNR, Bari, Italy

**Keywords:** parametric data analysis, bond lengths, polyhedral volumes, covariance, software, *Crystal Palace*

## Abstract

*Crystal Palace* is a new Windows program for Parametric Analysis of Least-squares and Atomic Coordination with Estimated standard uncertainties (e.s.u.’s). The primary purpose of the program is to organize the refined structures from parametric structural studies (as a function of pressure or temperature or a series of compositions) for analysis of the structural trends, and the production of tables for publication without the risks associated with manual editing. The program reads structural information from one or more crystallographic information format (cif) files. It organizes the data by finding the structurally equivalent atoms in each structure and therefore can correctly organize structural information even if atom names or site occupancies are different, or the atom lists in the cif files are ordered differently. A major shortcoming of cif files as currently used is that they do not contain the full variance–covariance matrix from the structure refinement, but only the uncertainties of the individual positional parameters. Without the covariance of positional parameters, the e.s.u.’s of bond lengths and angles cannot be determined. *Crystal Palace* uses symmetry to estimate the major contributions to the covariance of atomic coordinates and thus realistic uncertainties of bond lengths, angles and polyhedral volumes. *Crystal Palace* also calculates various polyhedral distortion parameters and rigid-body corrections to bond lengths.

## Abstract 2

*Crystal Palace* is a new Windows program for parametric analysis of least-squares and atomic coordination with estimated standard uncertainties. It allows the easy analysis of how crystal structures change with temperature, pressure or composition.

## 1. Introduction

Correct estimation of the uncertainties of structural parameters such as bond lengths, angles and polyhedral volumes is essential for the correct comparison of corresponding values from different structure refinements. A simple example would be whether a bond length or an intermolecular contact varies significantly and systematically in a parametric study as a function of pressure or temperature.

In order to correctly calculate the uncertainties in any structural property of a crystal structure, for example an interatomic distance, a bond angle or a polyhedral volume, it is necessary to have the full variance–covariance (VCV) matrix ***S*** of all of the positional parameters and the unit-cell parameters that contribute to the parameter being calculated. If we denote the calculated property as *p* and the *n* parameters (including the fractional coordinates of atoms and cell parameters) as *x_i_*, then the variance of *p* is given by
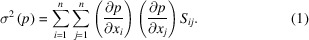


It is normally, and not unreasonably, assumed that the uncertainties in unit-cell parameters and the uncertainties in the fractional coordinates of the atoms are completely independent of one another. Then the contribution of these two sources of uncertainty to 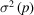 can be calculated separately through (1) and simply summed together.

The VCV matrix of the fractional coordinates includes separate contributions from different sources. There is the intrinsic variance of the positional parameters and the covariance between them that arises from the least-squares refinement of the parameters to the diffraction data. Beyond what is recorded in the VCV matrix from least-squares are two additional types of contributions from the constraints on fractional coordinates arising from the space-group symmetry of the structure. One is the 100% correlation of the atomic coordinates of a single atom that are constrained to be equal, such as *x* = *y* on a (110) mirror plane in tetragonal crystals. The other is the 100% correlation between the coordinates of atoms that are equivalent by the symmetry. Neither of these contributions involving symmetry appear in the VCV matrix from a least-squares refinement because the fractional coordinates generated by symmetry are not variables in a structure refinement. Nonetheless, refinement programs that calculate such derived structural variables have the full VCV matrix of the positional parameters from least-squares available as well as all of the symmetry information, and can therefore produce correct estimated standard uncertainties (e.s.u.’s, Schwarzenbach *et al.*, 1995 ▸) of calculated quantities. Tests indicate that *Rfine* (Finger & Prince, 1974 ▸) and *SHELX* (Sheldrick, 2008 ▸, 2015 ▸) are examples of refinement packages that do so correctly, but that *Jana2006* (Petříček *et al.*, 2014 ▸) does not allow for the correlation between atoms related by symmetry.

A major shortcoming of crystallographic information format (cif) files as currently used is that they do not contain the full VCV matrix from the structure refinement. Instead, only the uncertainties of the individual positional parameters and not their covariance are reported. The consequence is that while the e.s.u.’s of bond lengths and angles reported in cif files generated by refinement programs should be correct, a naïve calculation of their uncertainties from the e.s.u.’s provided in the cif file will be wrong because their covariance will have been assumed to be zero (*e.g.* Hazen & Finger, 1982 ▸; Parsons & Clegg, 2009 ▸; Schwarzenbach *et al.*, 1995 ▸). As a simple example, consider a structure in space group 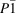 with a cation *M* at the origin, an O atom at 0.200 (2), 0.0, 0.0 and a unit-cell parameter *a* = 10.0 Å (with no uncertainty). This example is provided as datablock example1 in the cif file in the supporting information to this paper so that the reader can easily apply the following test to any program that calculates bond lengths and angles. The *M*–O distance and its e.s.u. can be correctly calculated as 2.00 (2) Å from the values given in the cif file. Because the O–*M*–O linkage is straight (by symmetry) then it is obvious that the O–O distance across the *M* is simply twice the *M*–O distance, and therefore by standard error propagation the O–O distance is 4.00 (4) Å. If we write out this calculation in a general way, then the O–O distance is
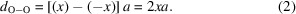
From which one again concludes correctly that 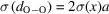. Equation (1) yields the same result, because the correct formula for uncertainty propagation in this case should involve the covariance 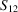 of the two fractional coordinates, thus
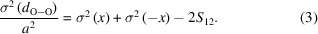


The correlation of *x* and −*x* is −100%, so 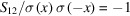 which makes 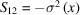. The full error propagation equation (3) derived from (1) then correctly gives 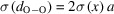.

However, many structure analysis and drawing programs and the *PLATON* package used for International Union of Crystallography structure validation (Spek, 2009 ▸, 2020 ▸) will report this distance as 4.00 (3) Å because they calculate the e.s.u. as 0.028 Å which is too small by a factor of 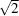. This is because the programs have assumed incorrectly that the uncertainties on the two coordinates of the two oxygens are independent of one another, and have applied the standard formula for the propagation of independent uncertainties that arises from (1) by assuming that *S*_12_ = 0:
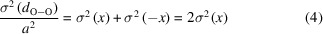
and thus 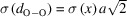.

While this might not seem to be a serious practical problem given that most cif files include the bond lengths and angles of primary chemical or physical interest, this is not always the case. For example, the intermolecular contacts in molecular crystals which control the packing are often not listed. In inorganic crystals the inter-ligand distances around cations are less frequently listed while anion–anion distances, which can control the response of framework structures to pressure (*e.g.* Angel *et al.*, 2012 ▸), are normally absent from cif files. The analysis of the response of inorganic structures in parametric studies is often focused on the evolution of cation–anion polyhedra and how their volumes change with pressure and temperature (*e.g.* Hazen & Finger, 1982 ▸; Hazen & Downs, 2000 ▸). Calculation of polyhedral volumes is rarely incorporated into refinement packages and reporting of polyhedral volumes is not supported by standard cif dictionaries, so these values must always be calculated post-refinement or post-publication with only the e.s.u.’s of refined parameters being available.

In this paper we show how the covariance between fractional coordinates can be estimated from the limited information given in a standard cif file. In the absence of the full VCV matrix from refinement this is not a complete solution, but it does yield more reasonable e.s.u.’s for structural parameters. This is because the correlation between positional parameters related by symmetry is, as noted above, 100% while the typical correlation between symmetry-independent parameters arising from least-squares is typically less (often much less) than 30%. The true values of the VCV matrix of the atomic coordinates are therefore dominated by the variances of the individual coordinates (the squares of the e.s.u.’s reported in the cif file) and their covariance induced by symmetry.

We introduce the freely available computer program *Crystal Palace* that implements these methods for the calculation of the e.s.u.’s of bond lengths, angles and polyhedral volumes from data provided in cif files, including cif files containing a series of parametric structure refinements. As far as we are aware, this is the first published program to fully calculate the e.s.u.’s of polyhedral volumes. The program also calculates the polyhedral distortion parameters introduced by Balić-Žunić (2007 ▸), Balić-Žunić & Makovicky (1996 ▸), Balić-Žunić & Vicković (1996 ▸), Makovicky & Balić-Žunić (1998 ▸), and includes several other features including riding motion corrections to bond lengths, and export utilities to other programs.

## 2. The solution

For convenience we can rewrite equation (1) in matrix form:
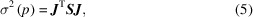
with ***S*** still the VCV matrix of the crystallographic parameters involved in the calculation. The ***J*** is a column vector whose elements are the derivatives 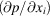. The general solution to the problem of calculating e.s.u.’s of derived parameters is to formulate the calculation of the parameters in a way that allows equation (5) to be applied unambiguously, and then to construct the appropriate VCV matrix ***S*** and calculate the vector ***J***. In the following sections we first discuss the specifics of the estimation of ***S*** and ***J*** and the calculation of 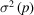, first from the uncertainties in the unit-cell parameters, and then from the uncertainties in the fractional coordinates.

### 2.1. The contribution of cell parameter uncertainties

For all calculations we make the common assumption that the values of the unit-cell parameters are completely uncorrelated with the values of the fractional coordinates of the atoms so that their contributions to 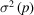 can be calculated separately through (5) and added together. The alternative approach of accommodating the uncertainties of the cell parameters by modification of the uncertainties in the fractional coordinates (Haestier, 2009 ▸) of the atoms is valid for the lengths of the cell edges, but cannot correctly allow for uncertainties of the unit-cell angles of monoclinic or triclinic crystals (Schwarzenbach, 2010 ▸). Therefore, matrix ***S*** for the contribution of the cell parameters is simply the VCV matrix of the cell parameters. In all cases, the diagonal elements *S_ii_* are the squares of the e.s.u.’s of the individual cell parameters, as provided in a cif file. Unit-cell angles whose values are fixed by symmetry have zero covariance with all other parameters. If the true covariance from the determination of the cell parameters is not known, a reasonable estimate of the off-diagonal elements *S_ij_* can still be made if the crystal system is known. If a pair of cell parameters have equal values by symmetry (*e.g.a* = *b* in uniaxial systems) then their correlation is 1 and their covariance *S_ab_* = 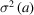 = *S_aa_* = *S_bb_*. The elements of the vector ***J*** are most easily calculated numerically by the brute-force method by adjusting each cell parameter in turn by a small amount, typically of the order of its e.s.u., and calculating the derivative from the resulting change in the value of the property. However, it is more computationally efficient to do the calculation in a more direct way by incrementing each cell parameter *i* in turn by its e.s.u., calculating a new metric tensor, and then calculating a new value of parameter *p*(*i*). The 100% correlation of cell parameters in crystal systems of higher symmetry (for example *a* = *b* in uniaxial crystals) is simply accommodated by incrementing the related parameters simultaneously by their e.s.u.’s. The contribution to 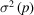 from all of the cell parameter uncertainties is then 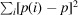 which is added to the variance of *p* resulting from the uncertainties in fractional coordinates.

### 2.2. Covariance of fractional coordinates of one atom

If an atom or point at **x**_0_ lies on a general equivalent position (g.e.p.) of the space group, then its *x*, *y* and *z* coordinates are not constrained by symmetry and the only covariance between them will arise from the least-squares refinement. In the absence of the VCV matrix from the refinement we have to assume that this covariance is negligible compared with the variance (squared uncertainties) of the individual coordinates. Under this assumption the VCV ***S***(**x**_0_) of a point **x**_0_ is simply diagonal with diagonal elements equal to the squares of the uncertainties.

The e.s.u.’s of the fractional coordinates of special equivalent positions (s.e.p.’s) that are fixed by symmetry should be reported correctly as zero in a cif file and they have zero covariance with the variable fractional coordinates. In space groups of symmetry higher than orthorhombic, some of the fractional coordinates of some s.e.p.’s are related to one another, for example *x* = *y* on a (110) mirror plane in tetragonal crystals. As for a g.e.p., the diagonal elements of ***S***(**x**_0_) are the squares of the individual e.s.u.’s of *x*, *y* and *z*; in the case of *x* = *y* then σ(*x*) = σ(*y*) and *S_xx_* = *S_yy_*. The off-diagonal components of ***S***(**x**_0_) which represent the covariances of the coordinates imposed by the symmetry of the s.e.p. can be deduced from the stabilizers of the s.e.p. which are the symmetry operators that leave the s.e.p. coordinates unchanged. If we denote the rotational part of a stabilizer as the matrix ***R*** that operates on the column vector **x**_0_, then the off-diagonal elements of ***S***(**x**_0_) are the off-diagonal elements of ***S***(**x**_0_)***R***^T^.

Calculations often involve a position 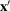 that is related by symmetry to the reported position **x**_0_ by a rotation ***R*** and a translation ***T***. It will have coordinates given by
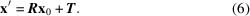


As the translational parts ***T*** of crystallographic symmetry operators are always fixed fractions, they do not contribute additional variance to the position 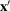, so the VCV matrix 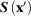 of the position 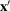 is obtained (Prince & Boggs, 1992 ▸) by
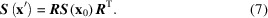


### 2.3. Covariance of fractional coordinates of two atoms

**Figure 1:**
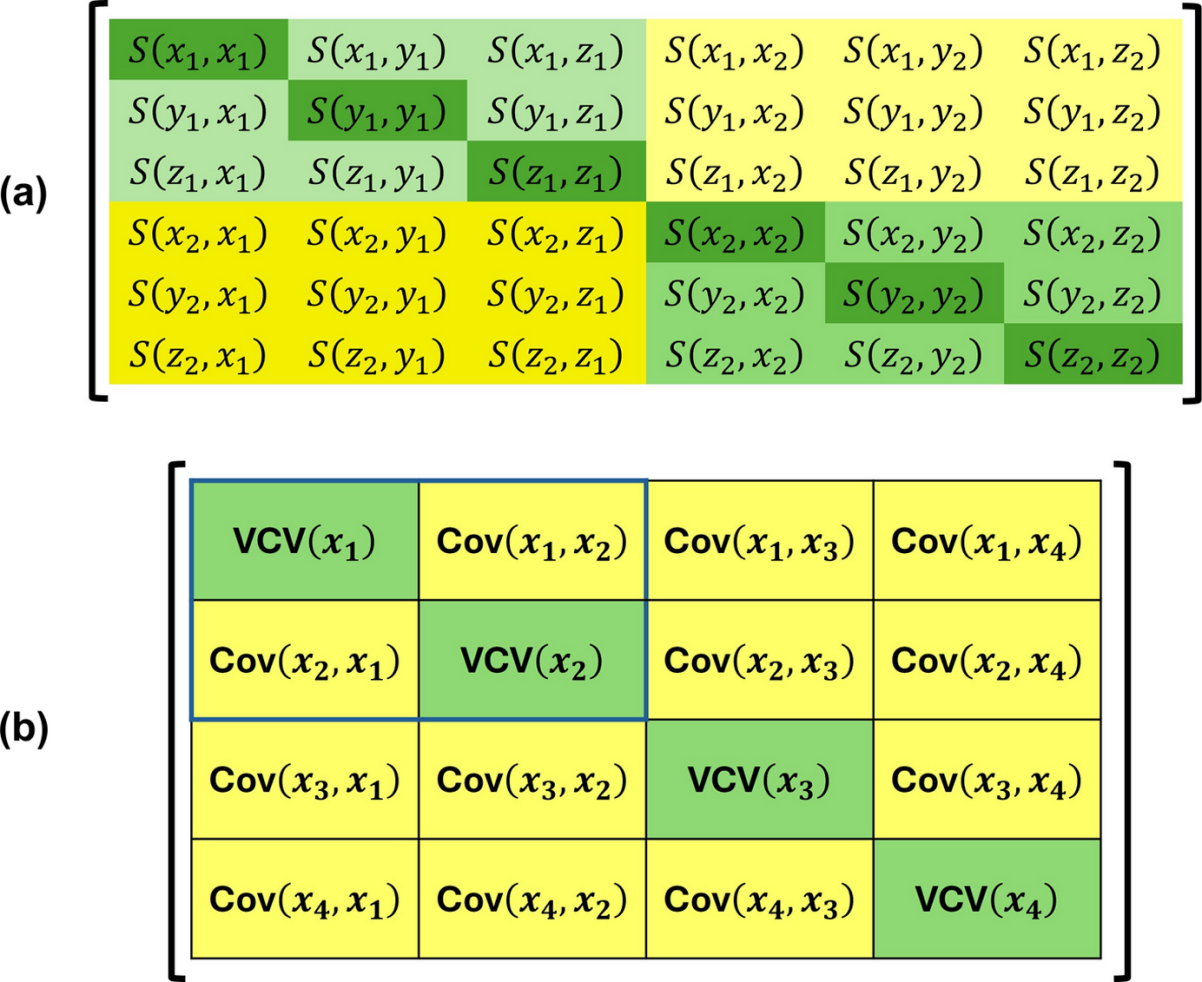
(*a*) A schematic representation of the elements of a VCV matrix ***S*** of the positional parameters *x*_1_, *y*_1_, *z*_1_ and *x*_2_, *y*_2_, *z*_2_ of two atoms. The diagonal elements marked in dark green, such as 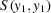, are the variances of the coordinates equal to the squares of the individual parameter e.s.u.’s quoted in cif files. The covariances of the positional parameters of a single atom are coloured in pale green. If the atom occupies a g.e.p. these elements are zero, unless the full VCV matrix is available from the least-squares refinement. If it occupies a s.e.p., for which the rotational part of the stabilizer is ***R***, then they are the off-diagonal elements of ***S***(**x**)***R***^T^ (Section 2.2). The covariances between the coordinates of the two atoms appear in the two yellow blocks. In the absence of the covariance from the least-squares refinement they are only non-zero if the atoms are related by symmetry, in which case their values are ***S***(**x**_1_)***R***^T^ where ***R*** is the rotational part of the symmetry operator relating the two positions. (*b*) A diagram showing the extension of the principle of construction of ***S*** as a 12 × 12 matrix for a calculation involving four atom positions, **x**_1_, **x**_2_, **x**_3_, **x**_4_, as required for calculating the volume of a tetrahedron. Each block in this diagram is a 3 × 3 matrix; the 3 × 3 blocks on the diagonal are the VCV matrices of the individual positions, and the off-diagonal 3 × 3 blocks are the covariances of individual pairs of atom positions. The area outlined in blue is the matrix elements shown in part (*a*).

The procedure described so far can be used to determine the individual VCV matrices 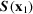 and 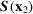 of two atoms or points at 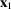 and 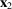. If these two points are completely independent of one another, are not related by symmetry and have no significant correlation between their values from least-squares, then the VCV is just the block-diagonal matrix constructed from 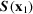 and 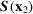, as illustrated schematically in Fig. 1 ▸(*a*).

When the positions **x**_1_ and **x**_2_ are related by symmetry their covariance as a 3 × 3 matrix ***S***′ = ***S***(**x**_1_)***R***^T^ can be calculated from the rotational part ***R*** of the symmetry operator that takes 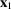 to 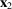. This matrix ***S***′ can then be inserted twice into the VCV matrix ***S*** of the entire calculation in the off-diagonal blocks corresponding to the positions of 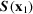 and 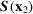, as shown in Fig. 1 ▸(*a*). If the parameter being calculated involves more than two atoms, for example three atoms to calculate an inter­atomic angle or four atoms to calculate the volume of a tetrahedron, the same method is applied to calculate the pair-wise correlation of the coordinates of each pair of atoms and to build the full matrix ***S***. This will have dimensions 3*n* × 3*n* for a calculation involving *n* atoms [Fig. 1 ▸(*b*)].

### 2.4. Calculation of e.s.u. of distances

If the vector between two atoms in a crystal structure with fractional coordinates 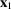 and 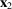 is 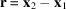 (all written as column vectors) then the distance *d* between the two points can be calculated with the metric tensor ***G*** of the crystal:
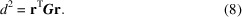
The uncertainty of a bond length 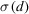 can be calculated via (5) using the 6 × 6 ***S*** matrix constructed from the e.s.u’s of 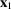 and 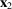 (Sections 2.2 and 2.3) and ***J***. However, unlike other derived structural parameters such as bond angles, equation (8) has a simple derivative with respect to the vector **r**. This allows 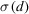 to be expressed in terms of the 3 × 3 VCV matrix 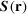 of the vector **r**:
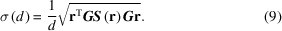
If the two points 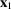 and 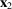 are completely independent of one another, then 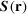 is simply the sum of the VCV matrices of the two end points:
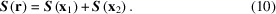
However, if the two end points of **r** are related by symmetry operation with rotation ***R*** and a translation ***T***, as in (6), then the vector from 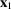 to 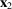 can be written as
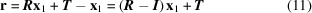
in which ***I*** is the identity matrix. The VCV matrix 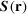 of the vector **r** is obtained by analogy to equation (7). Thus
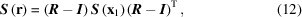
which can be used in (9) to correctly calculate the uncertainties in distances allowing for the correlation of positional parameters, including for the datablock example1 in the supporting information cif file.

### 2.5. Calculation of e.s.u. of angles

Calculation of interatomic angles involves the positions of three atoms, 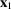 the central atom, and two positions 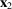 and 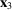. The angle θ_213_ at atom 1 between the bonds to atoms 2 and 3 is the angle between the vectors 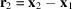 and 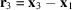 and is thus expressed in terms of the nine individual fractional coordinates of the atoms via the dot product between 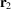
and 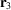:
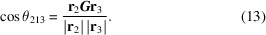
Unlike bond lengths, there is no simple useful derivative of this equation. Therefore the 9 × 9 covariance matrix between the three sets of positional coordinates is constructed as described in Sections 2.2 and 2.3. It is easiest to calculate the nine components of the ***J*** by the brute-force method (Hazen & Finger, 1982 ▸). Each fractional coordinate **x**_*i*_ involved in the calculation is shifted in turn by a small amount Δ*x_i_* and the value of the angle 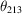 is recalculated. If the shift induced in the value of 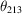 is 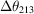 then 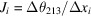.

The uncertainty on the angle θ_213_ then follows by application of (5). If the central atom lies on a s.e.p. then the coordinates 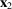 and 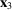 will be related by a stabilizer of the position 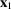 of the central atom (*i.e.* the symmetry operator that leaves 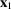 unmoved and transforms 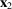 to 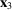). If the central atom lies on a rotation axis of order *n*, and all three atoms lie on a plane perpendicular to the rotation axis, then the value of θ_213_ will be constrained by symmetry to 360/*n* degrees and the correct value of the uncertainty is zero. For other cases when 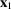 is on a s.e.p. the value of θ_213_ is not constrained by symmetry, but the calculation of its e.s.u. should include the covariance of the positions of 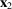 and 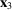. The datablock example2 (in the supporting information cif file) is provided as a test case with an O atom on a g.e.p. and an *M* atom at the origin in space group *P*1*m*1 giving an O–*M*–O angle of 82.70°. When the full VCV matrix is used in the calculation, the e.s.u. of the angle is 0.64° which, under the guidelines of Schwarzenbach *et al.* (1995 ▸), would be reported as 82.7 (7)°. If the covariance of the atom positions is ignored, as in many post-refinement calculation programs, then the incorrect e.s.u. = 0.45° will be obtained and the angle reported as 82.7 (5)°.

### 2.6. Calculation of e.s.u. of polyhedral volumes

A coordination polyhedron is defined by the positions of the ligands as its corners. As explained by Swanson & Peterson (1980 ▸), polyhedral volumes are calculated by transforming the ligand coordinates into a Cartesian space and dividing the polyhedron into non-intersecting component tetrahedra, each of which has three ligand positions as corners, with the fourth corner being a common internal point used for all of the component tetrahedra. Care has to be taken not to double-count volumes when more than three ligand positions are co-planar on one side of the central atom, thus forming an external face of the polyhedron with more than three corners. The method of determining the true external faces of the coordination polyhedron is exactly the same as used in building a crystal model for face-based absorption corrections (*e.g.* Burnham, 1966 ▸).

The volume of a polyhedron does not depend on the position of the central atom, so the uncertainty in a polyhedral volume depends only on the uncertainties and covariance in the positions of the ligands, and the uncertainties and covariance of the unit-cell parameters. The e.s.u. of each component tetrahedron can be calculated from the uncertainties in the coordinates of the ligands (Balić-Žunić, 2007 ▸). But each ligand position will be used in the calculation of several adjacent component tetrahedra, so the covariance of the volumes of the component tetrahedra is almost impossible to calculate. Therefore, the calculation of the e.s.u. of the polyhedral volume should proceed from the coordinates of the *N* ligands with the construction of the 3*N* × 3*N* VCV matrix of the coordinates of all the ligands, by the methods outlined in Sections 2.2 and 2.3. The ***J*** matrix is obtained by calculating the increment in polyhedral volume for small increments of each individual ligand coordinate, and the contribution of the ligand coordinate uncertainties to the variance of the polyhedral volume is obtained by applying equation (5), to which is added the variance due to the unit-cell parameters calculated as in Section 2.1.

Previous programs have either not calculated the e.s.u.’s of the volumes (*e.g.* Swanson & Peterson, 1980 ▸; Momma & Izumi, 2008 ▸; Spek, 2009 ▸, 2020 ▸) or have applied some approximations. The effect of these approximations is largest for coordination polyhedra with high point symmetries because these have the most ligands related by symmetry and thus the effect of the symmetry-induced covariance between the ligand coordinates will be greatest. As an example, we take the 8-coordinated polyhedron around the Zr atom in the structure of zircon, ZrSiO_4_, and use the room-pressure structure [this is the structure used by Hazen & Finger (1982 ▸) to illustrate the *Volcal* program] reported by Hazen & Finger (1979 ▸). The volume of the ZrO_8_ polyhedron is 19.004 Å^3^. The uncertainties and covariance in the unit-cell parameters contribute 0.003 Å^3^ to the e.s.u. of the polyhedral volume. The ZrO_8_ polyhedron has point symmetry 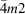 so that all eight O ligands are symmetrically equivalent. When the symmetry-induced covariance of the ligand positions is included along with the unit-cell covariance, the e.s.u. of the volume is 0.037 Å^3^. The *Volcal* program, written by Finger in 1971 and first published by Hazen & Finger (1982 ▸), uses the full covariance of the unit-cell parameters in calculations, but only the individual e.s.u.’s of the ligand coordinates to obtain a smaller e.s.u. of the polyhedral volume of 0.023 Å^3^. This again illustrates the common principle that e.s.u.’s of derived parameters increase when the covariance is included.

The *IVTON* program (Balić-Žunić & Vicković, 1996 ▸) uses a less intensive computation, by first calculating the e.s.u. of each component tetrahedron from the uncertainties in the unit-cell parameters and the ligand positions but without the covariance due to symmetry. Because each ligand position appears as a vertex in several adjacent component tetrahedra, the approximation is made that all of the component volumes are 100% correlated with one another, so that their individual e.s.u.’s are summed to obtain the e.s.u. for the polyhedron volume. This combination of approximations leads to an e.s.u. of the volume which tends to be slightly larger than the true e.s.u. for larger coordination numbers, for example 0.041 Å^3^ compared with 0.037 Å^3^ for the ZrO_8_ polyhedron in the zircon example. For polyhedra with lower point symmetries the assumption of 100% covariance in *IVTON* leads to e.s.u.’s of their volumes up to twice that calculated by using the covariance of the positional parameters (*e.g.* Table 1 ▸), although there are some exceptions such as the SiO_4_ tetrahedron in the same zircon structure, for which the approximation implemented in *IVTON* yields a smaller e.s.u. of 0.007 Å^3^ than the correct value of 0.011 Å^3^.

The physical reasonableness of the e.s.u.’s calculated by using the full covariance of the positional parameters and that of the cell parameters can be demonstrated in two ways. The volumes of some octahedra can also be calculated directly from the ligand–ligand distances forming the diameters of the octahedron. For example, an octahedron with *mmm* symmetry has three diameters of length *d*_*i*_ which are mutually perpendicular to one another, and the volume of the octahedron is
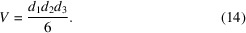
The uncertainty in the volume follows from (1), noting that 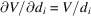:
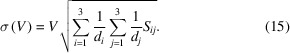
In this case ***S*** is the 3 × 3 VCV matrix of the three diameters, with the e.s.u.’s of each diameter calculated from the full covariance matrix of the cell parameters and the positional parameters of the two ligands, as described above in Section 2.4. If the octahedron is distorted from *mmm* symmetry, the true volume can be used as *V* in (15) to obtain an estimate of 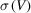. For octahedra of *mmm* symmetry or lower, the three diameters are independent of one another, and the off-diagonal elements of ***S*** are zero. When two diameters *i* and *j* are related by symmetry then their variances and their covariance are equal, 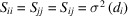. Table 1 ▸ shows that the e.s.u.’s of polyhedral volumes estimated in this way are very similar to the e.s.u.’s of the polyhedral volumes calculated directly from the full covariance matrix of the positional parameters of the ligands. The agreement is exact for octahedra in which the diameters are constrained by symmetry to be mutually perpendicular. When the diameters are not constrained to be perpendicular, most notably in the example of spinel, the additional angular degrees of freedom mean that (15) tends to underestimate the true 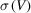.

**Figure 2:**
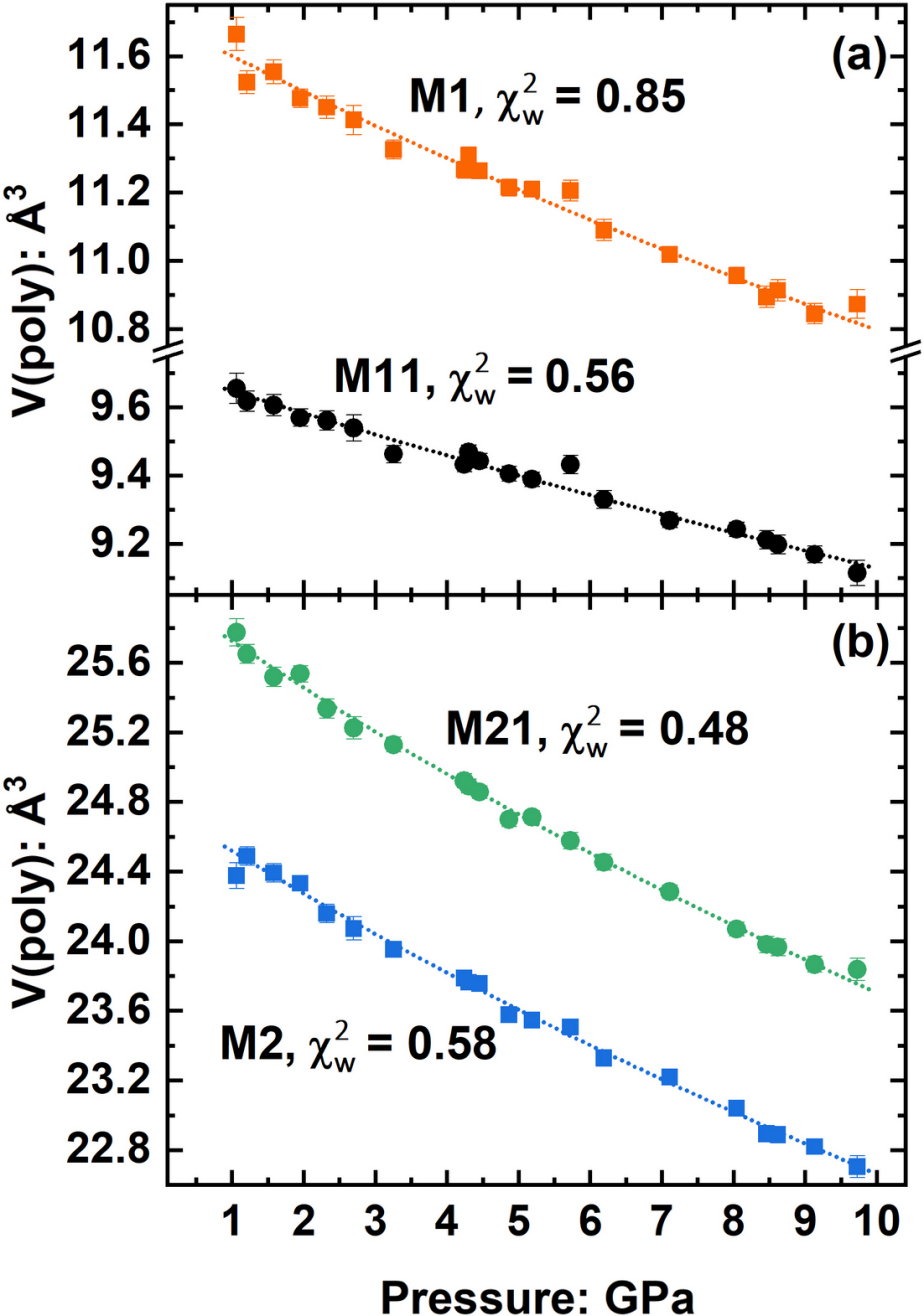
The variation with pressure of the volumes of four coordination polyhedra in a silicate pyroxene mineral (Baratelli *et al.*, 2025 ▸) calculated with the *Crystal Palace* program. If error bars are not visible, then the e.s.u. is smaller than the symbol size. The lines are second-order Birch–Murnaghan equation of state fitted to the data with *EosFit-GUI* (Gonzalez-Platas *et al.*, 2016 ▸) using both the uncertainties in pressure and the polyhedral volumes as weights, with the latter calculated in the *Crystal Palace* program using the full covariance of the ligand coordinates and unit-cell parameters. The values of 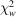 of the order of unity indicate that the e.s.u.’s calculated for these polyhedral volumes are realistic estimates.

**Figure 3:**
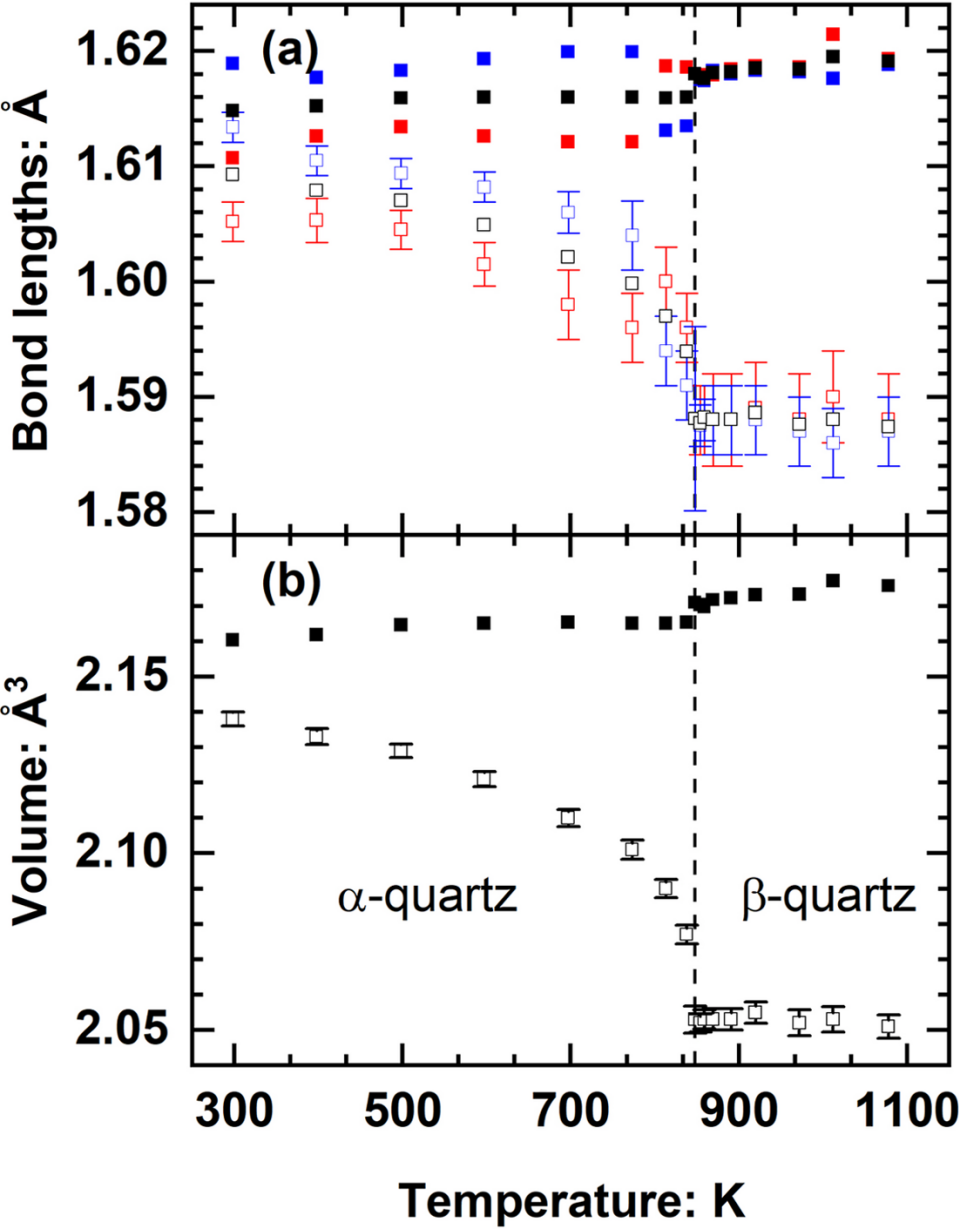
Variation of (*a*) the two independent Si–O bond lengths (red and blue symbols) and average bond length (black symbols), and (*b*) the SiO_4_ polyhedral volume in quartz as a function of temperature, all calculated with the *Crystal Palace* program from data of Kihara (1990 ▸). The open symbols are the lengths and volumes calculated from the reported refined parameters and they decrease unphysically with increasing temperature below the phase transition from α to β quartz at 848 K (dashed line). The bond lengths and volume corrected for thermal motion by the riding model (filled symbols) exhibit a weak positive thermal expansion.

The other criterion for evaluating the e.s.u.’s of any parameter from a parametric study is whether the e.s.u.’s of individual data reflect the overall data scatter from the general trend of the values. Fig. 2 ▸ shows the variation of individual polyhedral volumes of two octahedra and two 8-coordinated polyhedra in a clinopyroxene mineral studied as a function of pressure (Baratelli *et al.*, 2025 ▸). The scatter of the volumes is explained by the e.s.u.’s calculated using the full VCV matrix of the positional parameters of the ligands. As another example, Fig. 3 ▸ shows that the e.s.u.’s of the Si–O bond lengths of quartz as a function of temperature (Kihara, 1990 ▸) appear to be over-estimated compared with the data scatter, but the e.s.u.’s of the SiO_4_ tetrahedral volumes calculated with the full symmetry-induced covariance (there are two independent O atoms) appear realistic.

## 3. Implementation

The methods described above for estimating uncertainties in structural parameters have been implemented in a program that runs as a command-line program under Windows, named *Crystal Palace* for *Crystal Parametric Analysis of Least-squares and Atomic Coordination with Estimated standard uncertainties*. The program is written in standard Fortran and built on the crystallographic Fortran modules library *Crysfml* (Rodriguez-Carvajal & Gonzalez-Platas, 2003 ▸). The program has been developed over the past decade from a previous program, *cifreader*, that was originally written to explore structural evolution and polyhedral tilting in tetrahedral frameworks and was first released in 2012 (Angel *et al.*, 2013 ▸).

The key distinction of the *Crystal Palace* program is that it provides a platform to organize the refined structures from parametric studies for analysis of the structural trends, and the production of tables for publication without the risks associated with manual editing. To achieve this, the program can read in an unlimited number of multiple structures, either as multiple blocks in one cif file or from several cif files. The only requirement is that the structures are similar, with the same space group and the same atomic sites (although some sites may be vacant in some structures). This is achieved by the user first selecting one of the structures as the ‘reference structure’. The atom positions of the reference structure are then projected into the crystal metric of the other structures and the nearest atom is defined as the equivalent atom. This allows equivalent atom positions to be identified in different structures even if their site names or the elements occupying the equivalent sites are different, or the atom lists in the cif files are ordered differently, or symmetrically equivalent positions are listed for some structures. Atomic sites with mixed occupancies are accommodated automatically. This makes the program useful for analysing literature data on solid solutions as well as parametric studies of a single composition.

Individual atoms, types of atoms, or individual structures or groups of structures can be excluded from calculations if required without any editing of the original cif files, which always remain un-modified by the program. Once the equivalent atoms in each structure have been identified automatically on input of the cif file or cif files, bond lengths, angles and polyhedral parameters are calculated subject to a user-selected bond length range and user control over whether certain types of atom–atom contact (such as between cations) are to be included. All bond lengths and angles have their e.s.u.’s calculated by the methods described in Section 2. Extensive testing shows that the e.s.u.’s of bond lengths and angles calculated in this way do not differ significantly from those reported in cif files whose e.s.u.’s are calculated from the full VCV matrix of the least-squares refinement. For polyhedral volumes the method described in Section 2.6 using the full VCV matrix of the positional parameters of the ligands is implemented.

**Figure 4:**
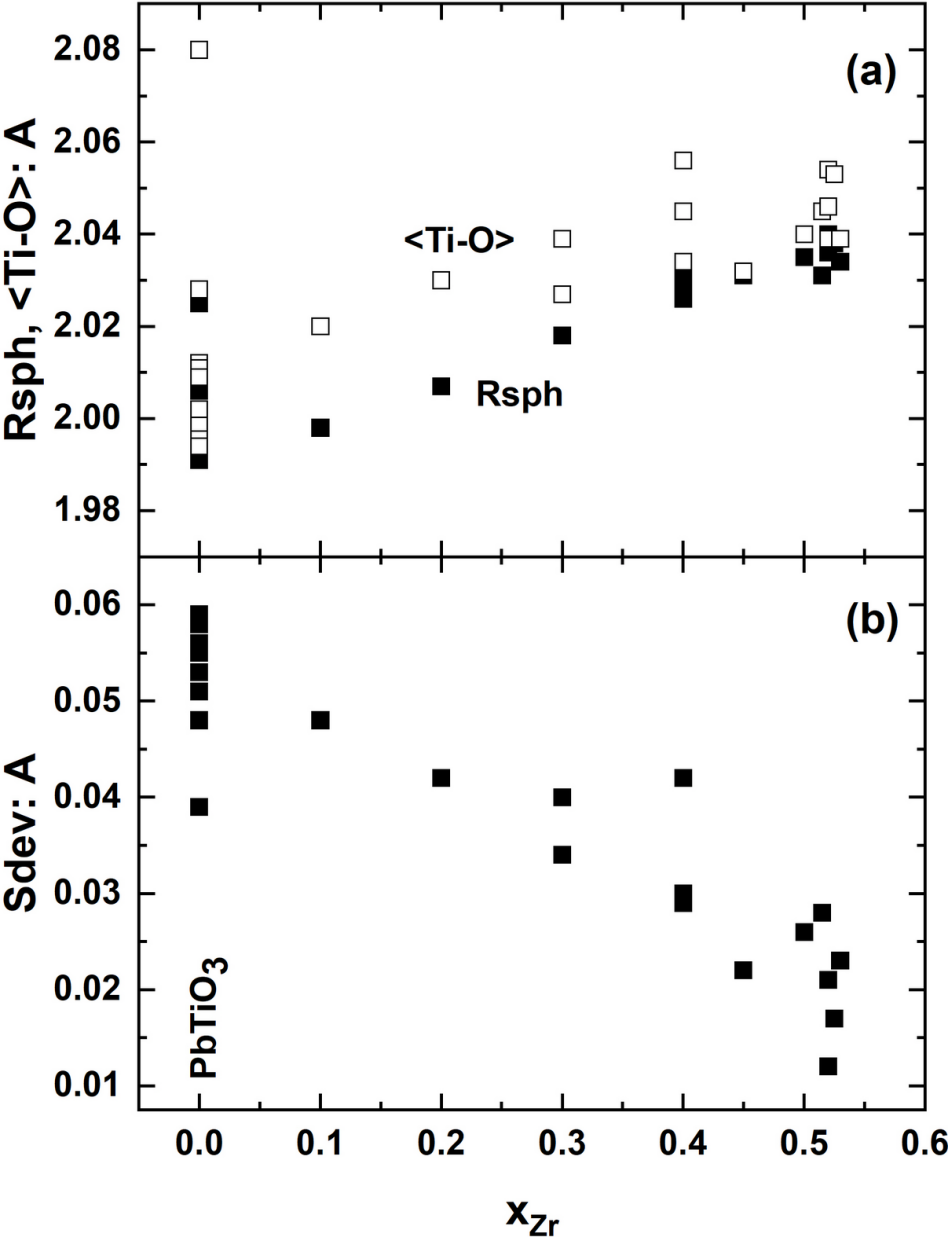
Structural evolution of the (Ti,Zr)O_6_ octahedron as Zr is substituted for Ti in the ferroelectric perovskite PbTiO_3_ to form PZT, from literature data. (*a*) The radius Rsph (Balić-Žunić, 2007 ▸) of the best fit sphere (solid symbols) shows much less data scatter than the average (Ti,Zr)–O bond length (open symbols), which in part depends on the refinement model used for the Ti,Zr site. (*b*) The Sdev parameter measures the spread of the distances of the O ligands from the centroid of the octahedron (Balić-Žunić, 2007 ▸). It shows that the configuration of oxygen atoms becomes more regular with increasing Zr substitution for Ti.

All output is organized by equivalent atoms, so that the parameters relating to the equivalent atoms or coordination polyhedra in the various structures appear together. Tables with e.s.u.’s are provided for fractional coordinates and anisotropic displacement parameters, transformed where necessary into the positions equivalent to that of the atoms in the reference structure. After calculation, tables with e.s.u.’s are available for bond lengths (including polyhedral volumes) and bond valences, angles and polyhedral edge lengths. Lists without e.s.u.’s are also provided for these parameters as well as site names, the polyhedral distortion parameters of Robinson *et al.* (1971 ▸) and Balić-Žunić and co-workers (Balić-Žunić, 2007 ▸; Balić-Žunić & Makovicky, 1996 ▸; Balić-Žunić & Vicković, 1996 ▸; Makovicky & Balić-Žunić, 1998 ▸). Fig. 4 ▸ shows an example of how these distortion parameters demonstrate details of the evolution of the (Ti,Zr)O_6_ octahedron as Zr is substituted for Ti in the ferroelectric PbTiO_3_.

The intramolecular bonds in molecules and the bonds in structures comprised of flexible frameworks of strongly bonded polyhedra are effectively rigid. As a consequence, raw bond lengths derived directly from the refined fractional coordinates do not represent the true bond lengths and can exhibit un-physical apparent thermal contraction (*e.g.* Fig. 3 ▸). This is a consequence of the correlated thermal vibrations of the strongly bonded atoms not being represented by the normal model of independent atomic vibrations used in standard structure refinements. When anisotropic displacement parameters are available for the atoms in the structure, *Crystal Palace* therefore can calculate the apparent displacement of atoms along all bonds (*e.g.* Hazen & Finger, 1982 ▸; Finger & Prince, 1974 ▸). Corrections to raw bond lengths and the corresponding polyhedral volumes are calculated using the ‘riding model’ implemented as the simple-rigid-bond calculation of Downs *et al.* (1992 ▸). After applying the riding model correction to the bond lengths and tetrahedral volumes of quartz, they exhibit the weakly positive thermal expansion expected for strong Si–O bonds (Fig. 3 ▸).

The *Crystal Palace* program includes utilities for modifying the data of either individual structures or groups of structures, for example site names or metadata such as temperatures or pressures, which are sometimes omitted from deposited cif files. The original cif files are not modified or overwritten, but new cif files can be written by the program. This allows the user to add such data to the cif files, or to add calculated bond lengths and angles if they were not present in the original cif file. Unit-cell parameters and calculated polyhedral volumes can be also written into a data file suitable for reading into the *EoSFit* program suite (Angel *et al.*, 2014 ▸; Gonzalez-Platas *et al.*, 2016 ▸) to fit equations of state.

The *Crystal Palace* program is available for free download from http://www.rossangel.com/home.htm and https://www.mineralogylab.com/ of the University of Pavia. Full documentation and instructional videos are provided through these websites. The program is not intended to be complete in its current form, but to provide a platform for the development of further parametric analysis of crystal structures.

## 4. Conclusions

The e.s.u.’s of structural parameters such as bond lengths and angles calculated by ignoring the covariance between atomic coordinates due to symmetry seriously underestimates their true values. The e.s.u.’s of bond lengths and angles calculated by including, as described here, the covariance due to symmetry do not differ significantly from those reported in cif files whose e.s.u.’s are calculated from the full VCV matrix of the least-squares refinement. This is because the covariances *S_ij_* between positional coordinates are proportional to their correlation coefficient *C_ij_*, which means that the value of the e.s.u. of a calculated parameter such as a bond length is increased by a factor of the order of 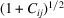 by correlation. The typical correlation between symmetry-independent parameters arising from least-squares is typically less (or much less) than 30%, or 0.3. Thus, even a correlation coefficient of 0.3 contributes only an additional 14% of the e.s.u. of a bond length that is calculated solely from the individual parameter e.s.u.’s, and this will often disappear in the reported e.s.u.’s which are always rounded up in value (Schwarzenbach *et al.*, 1995 ▸). On the other hand, the correlation between positional parameters related by symmetry is 100% and this can double the variance of a bond length, increasing the e.s.u. by more than 40%, as demonstrated in the *Introduction* by equation (3). We have also demonstrated that the extension of this method to the calculation of polyhedral volumes provides physically reasonable values of their e.s.u.’s. The method can be extended to any other structural quantity calculated from the refined positional parameters of atoms in a crystal structure.

## Supplementary Material

Crystal structure: contains datablock(s) CrystalPalace_example1, CrystalPalace_example2. DOI: 10.1107/S2053273325002682/ae5159sup1.cif

CCDC references: 2434032, 2434033

## Figures and Tables

**Table 1:** Validation of octahedral volume e.s.u.’s The e.s.u.’s listed were calculated as follows. *IVTON*: from the e.s.u.’s of the component tetrahedra, which are in turn calculated from the e.s.u.’s of the ligand coordinates without their covariance, and the unit-cell parameter e.s.u.’s. Full VCV: direct calculation from the full VCV of the ligand coordinates and the full covariance of the unit-cell parameters. Equation (15): calculated from the full VCV of the diameters. References and comments for these structures: 1: Baratelli *et al.* (2025 ▸), room-pressure datum. The evolution of these polyhedral volumes with pressure is shown in Fig. 2 ▸(*a*). 2: Angel *et al.* (2022 ▸). 3: Tetragonal perovskite, space group *P*4/*mmm*. All atoms on s.e.p.’s with fixed coordinates and e.s.u.’s of interatomic distances and polyhedral volumes only arise from the e.s.u.’s of the unit-cell parameters. 4: Zhao *et al.* (2009 ▸), room-pressure structure of 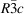 perovskite. 5: Unpublished structure. 6: Octahedron centred on origin of space group 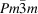, with ligands at 0.5, 0, 0. The e.s.u.’s of interatomic distances and polyhedral volumes only arise from the e.s.u.’s of the unit-cell parameters.

Structure	Reference	Site	Symmetry	Volume (Å^3^)	Diameters (Å)	σ*IVTON* (Å^3^)	σFullVCV (Å^3^)	σ[equation (15)] (Å^3^)
Clinopyroxene	1	*M*1	2	11.518	4.115 (7) ×2	0.060	0.042	0.044
					4.103 (7)			
Clinopyroxene	1	*M*11	2	9.534	3.851 (7) ×2	0.053	0.035	0.039
					3.859 (7)			
Olivine Ol9A	2	*M*2	*m*	12.423	4.2204 (15)	0.011	0.0060	0.0053
					4.2133 (7) ×2			
Olivine Ol9B	2	*M*2	*m*	12.407	4.2198 (7)	0.005	0.0028	0.0026
					4.2102 (4)			
					4.2104 (4)			
Olivine Ol9A	2	*M*1	1	11.848	4.1723 (15)	0.008	0.0076	0.0072
					4.1480 (14)			
					4.1736 (15)			
Olivine Ol9B	2	*M*1	1	11.827	4.1717 (7)	0.004	0.0034	0.0034
					4.1434 (7)			
					4.2715 (7)			
Tetragonal pv	3	*B*	4/*mmm*	7.024	3.470 (10) ×2	0.035	0.045	0.045
					3.500 (10)			
PrAlO_3_ pv	4	Al	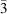	9.119	3.7966 (2) ×3	0.038	0.014	0.014
Spinel	5	*M*	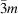	9.492	3.8712 (7) ×3	0.004	0.0067	0.0053
Cubic pv	6	*B*	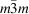	7.024	3.480 (10) ×3	0.035	0.061	0.061

## Data Availability

No original data have been used in this paper.
